# Supplementation with Flaxseed Oil Rich in Alpha-Linolenic Acid Improves Verbal Fluency in Healthy Older Adults

**DOI:** 10.3390/nu15061499

**Published:** 2023-03-21

**Authors:** Toshimi Ogawa, Kento Sawane, Kouta Ookoshi, Ryuta Kawashima

**Affiliations:** 1 Department of Advanced Brain Science, Institute of Development, Aging and Cancer, Tohoku University, Sendai 980-8575, Japan; ryuta.kawashima.a6@tohoku.ac.jp; 2 Smart Aging Research Center, Tohoku University, Sendai 980-8575, Japan; 3 Innovation Center, Central Research Laboratory, NIPPN Corporation, Atsugi 243-0041, Japan; ksawane14@gmail.com (K.S.); k-ookoshi@nippn.co.jp (K.O.)

**Keywords:** alpha-linolenic acid, cognitive function, RCT, age, flaxseed oil

## Abstract

The effects of docosahexaenoic acid supplements on cognitive function have long been demonstrated, but the effects of alpha-linolenic acid, a precursor of docosahexaenoic acid, have not been fully tested. The search for functional foods that delay cognitive decline in the older adults is considered a very important area from a preventive perspective. The aim of this study was to conduct an exploratory evaluation of alpha-linolenic acid on various cognitive functions in healthy older subjects. Sixty healthy older adults aged 65 to 80 years, living in Miyagi prefecture, without cognitive impairment or depression, were included in the randomized, double-blinded, placebo-controlled clinical trial. Study subjects were randomly divided into two groups and received either 3.7 g/day of flaxseed oil containing 2.2 g of alpha-linolenic acid, or an isocaloric placebo (corn oil) containing 0.04 g of alpha-linolenic acid for 12 weeks. The primary endpoints were six cognitive functions closely related to everyday life: attention and concentration, executive function, perceptual reasoning, working memory, processing speed and memory function. After 12 weeks of intake, changes in verbal fluency scores on the frontal assessment battery at bedside, a neuropsychological test assessing executive function, in which participants are asked to answer as many words as possible in Japanese, were significantly greater in the intervention group (0.30 ± 0.53) than in the control group (0.03 ± 0.49, *p* < 0.05). All other cognitive test scores were not significantly different between the groups. In conclusion, daily consumption of flaxseed oil containing 2.2 g alpha-linolenic acid improved cognitive function, specifically verbal fluency, despite the age-related decline, in healthy individuals with no cognitive abnormalities. Further validation studies focusing on the effects of alpha-linolenic acid on verbal fluency and executive function in older adults are needed, as verbal fluency is a predictor of Alzheimer’s disease development, important for cognitive health.

## 1. Introduction

In Japan, a super-aged society, the number of patients with dementia is increasing, and it is estimated to exceed 7 million by 2025 [1]. Dementia not only causes physical impairment, social impairment and a decline in the quality of life for the individual, but also places a heavy burden on family caregivers. This has developed into a social problem, as caregivers have fallen into a state of mental and physical exhaustion [2]. In some cases, this may lead to acts of violence against older adults, and vice versa [3,4]. Although much research has been conducted into the mechanisms and treatment of dementia, no fundamental cure has been found so far, and the importance of preventing dementia and alleviating its symptoms is being emphasized.

A growing body of research is showing that nutrients in foods have a positive impact on brain health maintenance suggesting an important role of nutrition in prevention. For example, the effects of diets containing docosahexaenoic acid (DHA), an ω-3 polyunsaturated fatty acid (PUFA) contained in fish oil, on cognitive function are becoming evident. According to a large meta-analysis, higher fish consumption is related to lower dementia risk [5] and a longitudinal study of more than 20 years of follow-up indicated the association between fish intake and cognitive function [6]. Moreover, Boespflug et al. showed in an intervention study that fish oil supplementation increased the bioavailability of DHA and blood supply to the posterior cingulate cortex, which resulted in increased working memory performance, suggesting the possibility of nutrient intervention on cognitive performance improvement [7], despite the decline in DHA levels with age. DHA is found in breast milk, and is necessary for brain development and directly involved in neurological function as an important component of neuronal cell membranes throughout life. Therefore, DHA consumption is required throughout life.

In addition to the direct inoculation of DHA, DHA precursors can be supplied as a means of promoting DHA synthesis in the body [8]. Alpha-linolenic acid (ALA) is another ω-3 fatty acid, known as a precursor that can synthesize DHA in the body, and is a component of edible oils, such as flaxseed oil and perilla oil. The mechanism of action is beginning to be clarified, as further evidence indicated that ALA inoculation increased DHA concentration in the rodent brain. ALA administration increases brain-derived neurotrophic factor (BDNF) and acts on neural elongation [9,10]. Although the efficiency of DHA synthesis is limited, the conversion rate of ALA to EPA in humans is approximately 0.1–21% of ingested ALA, and the conversion rate to DHA is 0.1–9% [11,12], and ALA can act similarly to DHA [13].

Human studies have shown that consumption of perilla oil (equivalent to 4.3 g/day of ALA) for 12 months, assessed by the frontal assessment battery at bedside (FAB), showed changes in cognitive function after the test, compared to pre-intervention [14]. Studies have also shown that a 1 g/day increase in the intake of ALA reduces mortality from myocardial infarction by 10% [15]. This suggests that as little as 1–4.3 g of ALA intake can exert a health function. Therefore, in the present study, 2.2 g/day of ALA was used. This is approximately twice the intake of omega-3 fatty acids in the Japanese population [16], but within the normal range, and is also based on the fact that the adequate intake of omega-3 fatty acids in the Japanese population is 2.1 g/day for men and 1.8 g/day for women over 75 years of age [17].

Thus, although dietary ALA may affect cognitive function positively, we considered that there is little evidence for an objective ALA intervention effect and this needs to be more clarified. Therefore, we decided to conduct a randomized controlled trial to evaluate ALA in healthy older adults, in an exploratory manner, on various cognitive functions. Flaxseed oil containing high concentrations of ALA (60% of the fatty acid composition) was determined to be a suitable source of ALA. Assessment of cognitive function focused primarily on attention and concentration, executive function, perceptual reasoning, working memory, processing speed and memory function.

## 2. Materials and Methods

### 2.1. Study Design

This intervention study was approved by the ethics committee of the Tohoku University Graduate School of Medicine on 23 March 2020 (Receipt number: 2019-1-957). Written informed consent was obtained from all subjects at the time of enrollment. The study was a 12-week randomized, double-blinded, placebo-controlled clinical trial among healthy regional older adults in Miyagi prefecture, Japan. The primary outcome of the study was the cognitive function, which was evaluated individually in-person at the Tohoku University. This RCT was registered at the University Hospital medical information network (UNIM) Clinical Trial Registry (UMIN000039901).

### 2.2. Participants

The local widely-read newspaper (Kahoku weekly) was used for subject recruitment, and 91 women and men living in the Miyagi prefecture, aged 65–80 years, visited the test center for screening. Sixty subjects were recruited according to the inclusion and exclusion criteria. The study was targeted at right-handedness, in order to reduce variations in cognitive ability due to the differences between right- and left-handed individuals [18]. In-person, participant-to-tester interviews were conducted using Mini-Mental State Examination (MMSE) and clinical dementia rating (CDR) to screen cognitive impairment, and using the geriatric depression scale (GDS) to screen mental status together with descriptive questions. An MMSE score of 27 and higher was set as a threshold to avoid participants with mild cognitive impairment. The sample size was set as the criterion for sample size adequacy, which can produce significant differences in cognitive psychological tests based on previous study [19]. 

### 2.3. Inclusion and Exclusion Criteria

#### 2.3.1. Eligibility Criteria

1. MMSE score of 27 and higher.
2. CDR score of 0.
3. Age: 65 or older, 80 or younger (at the time of registration).
4. Right-handedness.

#### 2.3.2. Exclusion Criteria

1. Participants with a history of severe cranial nerve disease or internal diseases.
2. Participants with severe visual or hearing impairment.
3. Extremely obese/thin (BMI less than 17 kg/m^2^ and more than 30 kg/m^2^).
4. Participants who have continuously consumed foods containing horse-mackerel type of fish.
5. Frequent fish consumers (3 or more servings/week as a staple food).
6. Participants with psychosis or psychiatric symptoms that make it difficult for them to participate in the study.
7. Other subjects the principal investigator considered difficult to participate in the study.

### 2.4. Randomization

Sixty selected subjects were randomly divided into two groups and supplemented with 3.7 g/day of flaxseed oil containing 2.2 g ALA (ALA group), or iso-calorie placebo (corn oil) (CONT group) containing 0.04g ALA for 12 weeks. The test sample allocation table was generated randomly, and study subjects were assigned. The study was blinded to both participants and researchers. 

### 2.5. Experimental Food and Study Settings

Flaxseed oil was provided by NIPPN Corporation (Tokyo, Japan), and corn oil was purchased from J-OIL MILLS, Inc. (Tokyo, Japan), and both were individually packed without any labels. The packages were completely identical in color, shape, and size between the test foods. Compliance was monitored by having the subjects mark the calendar provided by the test center whenever the test food was consumed and asking them to bring the calendar with them when they visited the study center for assessments during the experimental period (pre-, after 6 and 12 weeks of intervention). Subjects were asked not to change their daily habits, including physical activity, diets, and daily behavior during the experimental period, and were free to take medications. Dietary habits were checked using the validated food frequency questionnaire (FFQg Ver. 5) [20], Japan, to ensure that there were no changes in diet, particularly in the intake of ω-3 fatty acids, during the study period. In addition, subjects were instructed to use the test oil as table oil during meals to avoid misuse, such as overheating the oil during cooking before ingestion. 

### 2.6. Subject Background Information Collections

The background data included the date of birth, comorbidities, medications treated, blood pressure, medical history (including allergies), and lifestyle information (smoking, alcohol consumption, food or dietary supplements and health food consumption). Height and weight were measured physically by a tester, and the body mass index (BMI) was calculated by dividing weight by height squared. BMI can be used as a screening for weight categories, as it correlates moderately with more direct measures of body fat [21,22].

### 2.7. Assessment of Cognitive Function

To explore a broad range of cognitive functions, there were six broad categories, all of which were closely associated with daily life activities: attention and concentration, executive function, perceptual reasoning, working memory, processing speed and memory function. All tests proceeded individually with two trained testers, who are research assistants at the Tohoku University (one tester for screening procedure, another for assessments pre-, after 6 and 12 weeks of intervention). All of the timing and details are described below.

Overall cognitive status was measured by MMSE and the Montreal cognitive assessment, MoCA-J, at pre- and after 12 weeks of intervention period. Attention was measured by digit cancellation task (D-CAT). Executive functions were measured by frontal assessment battery at bedside (FAB) and Stroop test. Intelligence was measured using WAIS-IV subscales and assessing in perceptual reasoning using block design (BD), matrix reasoning (MR) and visual puzzles (VP). Working memory was measured by digit span (DS) and arithmetic (AR). Processing speed was measured by coding (CD) and symbol search (SS). The short-term memory was assessed by visual and verbal memory tests, attention and concentration were assessed by visual memory range and digit span and using the subscale of WMS-R. The summary of the assessment tasks is presented in Table 1 and the details of all tasks are described below. 

#### 2.7.1. Mini-Mental State Examination

The MMSE is the most widely used dementia screening test in the world, which detects cognitive impairment in older adults in short time (5–10 min), but is not timed. The MMSE covers orientation, memory and attention, and tests the ability to name, follow verbal and written commands and write a sentence spontaneously [23]. Its score vary with age and education [24]. The MMSE is scored from 0 to 30. Lower MMSE scores indicate more impairment. A total score of 27 or higher is considered within the normal range without possible MCI, which ranges between 23 and 26. The score was not adjusted for age and education.

#### 2.7.2. Montreal Cognitive Assessment

The MoCA is a screening test with a high ability to discriminate normal cognitive function, and MCI and early onset dementia, providing quick guidance for referral and further investigation of MCI. It provides a comprehensive cognitive test and is designed to assess executive functions, higher-level language abilities, and complex visuospatial processing [25]. An MoCA score of 26 or higher out of 30 is considered within the normal range, and the test takes approximately 10 min to complete, but is not timed.

#### 2.7.3. Frontal Assessment Battery at Bedside

The FAB [26] assesses executive function. The FAB consists of six subtests exploring the following: conceptualization, mental flexibility, motor programming, sensitivity to interference, inhibitory control and environmental autonomy. The FAB is rated on a scale of 0–18, with lower FAB scores indicating a higher degree of executive dysfunction.

#### 2.7.4. Stroop Test

This test is to answer the reading of the word and the color name of the ink. First, participants practice once, then do this trial four times. They choose the ink the words represent. Second, the combination of words and ink colors are mismatched, but the ink that the words represent is chosen by not being distracted by the color of the ink. Third, the word that corresponds to the color of the ink is chosen. Fourth, the combination of words and ink colors are mismatched, but the word that corresponds to the color of the ink on which the words are written is chosen.

#### 2.7.5. Digit Cancellation Task

The D-CAT assesses attention. The test form consists of 12 rows of 50-digit numbers. Each row contains five random pairs of numbers from 0 to 9. Thus, one number appears five times in each row and its neighbor is determined randomly. The D-CAT consists of three such sheets. Participants were instructed to locate the designated target number and remove each number with a slash mark as quickly and as accurately as possible, until the experimenter gave a stop signal. One minute was given for each trial, so the D-CAT took a total of three minutes. On the second and third trials, the emphasis was on cancelling all indicated target numbers without omission. The main indicator for this test was the number of hits (correct answers); only the number of hits was used in the first trial.

#### 2.7.6. Wechsler Adult Intelligence Scale

##### Coding 

Coding is a sub-test of WAIS-IV. This test measures processing speed. For Cd, the participants were shown a series of symbols paired with numbers. Using a key, the participants drew each symbol under its corresponding number within a 120 s time limit. The primary measure of this test is the number of correct answers. 

##### Symbol Search

SS is a sub-test of WAIS-IV. This test measures processing speed. The SS consists of 60 items. In this test, participants visually scanned two groups of symbols (target group and search group) and indicated whether one of the symbols in the target group matched one of the symbols in the search group. Participants answered as many items as possible within a time limit of 120 s. The main measure of this test is the number of correct answers.

##### Digit Span 

Working memory was assessed with a three-digit span task. A series of digits were read, either in the same order, in reverse order or by rearranging the digits in ascending order.

##### Arithmetic 

Subjects responded to verbally presented arithmetic statements by mental arithmetic within a time limit. The test concerns mental operations, concentration, attention, short-term and long-term memory, numerical reasoning ability and mental agility, and continuous processing, fluid reasoning, quantitative reasoning, logical reasoning and quantitative knowledge. 

##### Block Design

Subjects were to make the same pattern as the model pattern presented to them using building blocks, within a time limit. The blocks consisted of a red and white two-color pattern.

##### Matrix Reasoning

Matrix reasoning consists of choosing the most appropriate option to complete the presented incomplete matrix from the choices.

##### Visual Puzzles

Participants selected the three pieces that, when combined, formed the same diagram as the sample, within a time limit.

### 2.8. Wechsler Memory Scale-Revised

The Wechsler Memory Scale-R (WMS-R) is a comprehensive test that assesses verbal memory, visual memory, general memory and attention and concentration functions. The verbal memory test was analyzed as the sum of logical memory and verbal versus associative, the visual memory test as the sum of graphic memory, visual versus associative and visual recall, and general memory function (short-term memory) as the sum of verbal and visual memory test results. The attention/concentration was analyzed by summing the mental control, digit span and visual memory span score. Each subtest was used according to the instruction (WMS-R David Wechsler).

### 2.9. Analysis

There was no exclusion or dropout during the study, therefore, all participants who were selected during the screening were entered in the statistical analysis. Statistical analyses were performed using IBM SPSS ver.24, and the differences between the ALA group and CONT group were analyzed by an unpaired t-test. The points of change in cognitive function before and after 6 and 12 weeks of evaluation were calculated. All data are expressed as the means ± standard deviations. Significance is inferred if *p* < 0.05. 

## 3. Results

**Figure 1:**
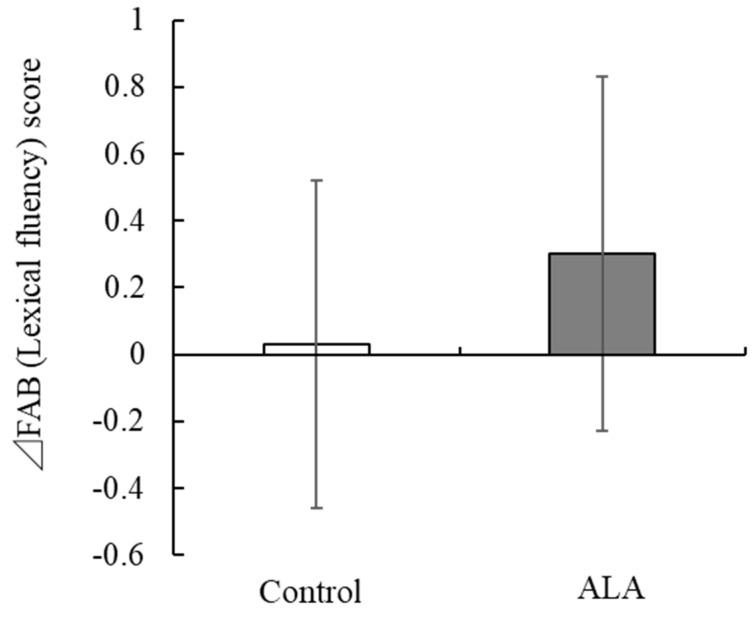
Effect of ALA intake on FAB (Lexical fluency) scores. Mean changes (∆) in the FAB (Lexical fluency) scores after 12 weeks of intervention in the ALA and control groups. Bar represents the mean ± SD. ALA, alpha-linolenic acid; FAB, frontal assessment battery.

A clinical trial was conducted to evaluate the functionality of daily consumption of flaxseed oil containing ALA on various cognitive functions. Sixty subjects were selected based on the inclusion and exclusion criteria and participated in the study of 91 screened participants. The study participant demographics and the intake of PUFA based on FFQ are presented in Table 2. It was revealed that several pre-intervention cognitive function scores were statistically different between the groups (Table 3), therefore, all the cognitive function scores were analyzed based on the change score (change between post-intervention score and pre-intervention score) for each of cognitive test. Precisely, the change scores were calculated between pre and 6 weeks after, and pre and 12 weeks after the intervention. After 12 weeks of intake, the change in verbal fluency score, a test in which participants were asked to answer as many words as possible beginning with a particular letter, such as “ka” in the Japanese language (Phonemic fluency [27]), on the FAB, a neuropsychological test that evaluates executive function, was significantly greater in the ALA group (0.30 ± 0.53) than in the control group (0.03 ± 0.49, *p* < 0.05) (Table 3, Figure 1). There were no significant differences in all other cognitive function test scores between the groups. 

## 4. Discussion

The purpose of our study was to explore the effect of 12-week daily consumption of flaxseed oil containing 2.2 g of ALA on various cognitive functions in healthy older adults without cognitive impairments, living in the community. The results of this study showed that consumption of flaxseed oil, which is high in ALA contents, improves cognitive function in healthy older adults. In particular, the results indicated positive improvements in the verbal fluency performance. While vocabulary and other language functions are thought to remain relatively unchanged with age, verbal fluency is known to decline with age and may interfere with conversation with others. In this study, verbal fluency performance was assessed as part of the FAB, which evaluates comprehensive executive function of frontal lobe function. The executive function is a higher-level function of the verbal fluency task [28] and is recognized as one of the key factors associated with the ability to set goals, make plans, modify and adjust while actually performing the actions, and carrying out effective actions in everyday life [29]. Thus, it is related to the ability of daily living/instrumental activities of daily living (ADL/IADLs) [30,31], social frailty [32] and importantly, life satisfaction [33] for older adults. In fact, such a simple verbal fluency task has a rather complex mechanism, as many cognitive functions are interrelated during this task, including semantic memory, dialectical lexical retrieval, information processing speed, inhibition, working memory, shifting performance and cognitive flexibility [29], and it includes a variety of anatomical sites. There are several possible explanations for the effects of ALA in the present study. As suggested in a previous review of the effects of DHA, it may have improved the efficiency of cognitive strategies, which decline with increasing age, by altering the structure of neuronal cell membranes in the broad anatomical regions and improving the fluidity and intercellular connectivity of the neuronal cell membrane, which declines with increasing age [34]. Examples of cognitive strategies include clustering (word generation) and switching (transfer of attention from one subcategory to another) strategies needed in verbal fluency tasks, both which are indeed said to be influenced by age [35,36] and implicated in Alzheimer’s disease [37]. Taken together, as a possible mechanism of the improved verbal fluency task, ALA was effective in a wide range of brain regions, acting broadly on neuronal structures and improving neuronal function from a cellular physiological perspective.

ALA is the most commonly consumed PUFA in Japan, with a median PUFA intake of 2.09 g/day for men and 1.83 g/day for women aged 75 years and older [16]. The dietary intake of PUFA during the intervention period in both groups of study participants was only slightly higher than that of the general population and, furthermore, there were no differences between the groups, so the PUFA intake of the participants was not considered to influence the study results. In addition to this, a distinction was made between ω-3 and ω-6 and analyzed pre- and post-intervention, but there were no differences between the groups. Comparatively, the conversion of ALA to DHA in the liver is reported to be inefficient [11,12], but the ALA intake, in this case taken daily, may have had a possible accumulative effect [38] and a small amount may have been sufficient [9,39]. This view is consistent with the consideration that DHA synthesis from ALA in humans is nutritionally adequate, despite the low rate of ALA-to-DHA conversion, as reported in a recent review [13]. Furthermore, in this exploratory study on various cognitive functions, ALA intake had no effect on anything other than the verbal fluency task. Considering the duration of the study, the age of the subjects and the sensitivity of the cognitive tests, it is possible that the decline over time in various cognitive functions was less detectable. 

Compliance with the intake of the test substance was high during the period of this study, which may have had a positive effect on cognitive function. One of the factors that contributed to maintaining high compliance was the use of pouch containers. The pouch container was light-shielding and had the advantage of being packaged in single servings, which could be left on the table and carried on the go, making it suitable for preventing forgotten intake and maintaining quality. According to the subjects, the oil itself was tasteless, odorless, easy to use, and compatible with meals, so it had the same level of usability as salt and pepper in a meal. We believe that in this study, we were also able to create a mechanism to encourage continued intake.

While the results of this study are encouraging, some limitations should be mentioned. Ideally, the recruitment of subjects should be unbiased to the general public when targeting community-dwelling older adults, but the recruitment was limited in some areas to newspaper readers, as opposed to internet users, because newspapers were used for this recruitment. It is also possible that the subjects may have been biased, as those with a particular interest in health, for example those who perceive a benefit from cognitive function tests, may have participated. Primary care centers and family physicians and researchers properly selecting the group of participants is more appropriate. To increase the generalizability of the study’s findings, it is necessary to diversify the target population in future studies. Various races and cultures also need to be considered. Second, although this study was exploratory in nature and subjects were asked to consume the test product daily in order to test its efficacy, the frequency, amount, and duration of consumption should be considered to accommodate a variety of dietary habits. Furthermore, since the effect on verbal fluency detected in this study was only detected using one item, a subscale of the FAB, it is necessary to use more specialized indexes related to verbal fluency in order to establish further evidence, and to increase the sample size, as this experiment is still at the preliminary research stage.

Regarding the impact of the results of this study, the importance of maintaining and even extending one’s verbal fluency capacity is one of the most important functionalities for older adults to age healthily. Many people, and the general public are unaware that some cognitive functions do not decline with age. Even the elderly, if they learn, should be able to increase their knowledge and vocabulary, help social interactions with others, and possibly form societal roles. In this context, if ALA intake can support verbal fluency even with increasing age, which is important for communication with others, it could promote social participation among older adults.

In summary, this RCT, evaluating the functionality of daily consumption of flaxseed oil containing 2.2 g of ALA, improved verbal fluency in healthy individuals aged between 65 to 80 years, with no abnormalities in cognitive function, despite declines with age. Since the improvement in verbal fluency is significant in cognitive health, as it is one of the factors of Alzheimer’s disease progress [37], further validation studies, with a focus on ALA’s impact on verbal fluency and executive function, are needed to extend the obtained evidence in this study to older adults.

## Figures and Tables

**Table 1:** Cognitive function tests.

Cognitive Functions	Task
Global cognitive status	Mini-Mental State Examination
	Montreal Cognitive Assessment
	Frontal Assessment Battery at bedside
Executive function	Stroop Test
Attention	Digit Cancellation Task
Intelligence	Wechsler Adult Intelligence Scale
Processing speed	Coding
	Symbol Search
Working memory	Digit Span
	Arithmetic
Perceptual reasoning	Block Design
	Matrix Reasoning
	Visual Puzzles
Memory	Wechsler Memory Scale Revised
Short-term memory	Verbal Memory: logical memory and verbal-paired associate
	Visual Memory: figural memory, visual-paired associates and visual reproduction
	General Memory: verbal and visual memory
Attention and concentration	Mental control: digit span and visual memory span

**Table 2:** Characteristics of the participants.

	Control		ALA		
	Means	SD	Means	SD	*p*-Value
Number of subjects	30		30		
Gender, % female	53.3		46.7		
Age(years)	72.1	4.3	71.9	3.7	0.411
Height(m)	1.6	0.1	1.6	0.1	0.813
Body weight(kg)	57.0	9.3	58.9	7.5	0.400
BMI(kg/m^2^)	22.4	2.5	23.1	2.1	0.273
ω-3 PUFA intake(g/day)					
pre-intervention	2.16	0.72	2.35	0.87	0.354
post-intervention	2.50	0.91	2.54	1.00	0.852
ω-6 PUFA intake(g/day)					
pre-intervention	10.80	3.15	10.34	3.33	0.587
post-intervention	11.49	3.76	11.23	4.06	0.803

**Table 3:** The scores of changes in cognitive functions.

	Baseline			Week 6		Week 12		Week 6—Baseline			Week 12—Baseline		
	Control	ALA	*p*-Value	Control	ALA	Control	ALA	Control	ALA	*p*-Value	Control	ALA	*p*-Value
MMSE													
Total score	29.27 (0.83)	28.77 (1.01)	0.04	-	−	28.33 (1.90)	27.97 (1.97)	−	−	−	−0.93 (2.13)	−0.80 (2.01)	0.084
MoCA													
Total score	24.47 (1.72)	23.40 (1.85)	0.024	-	−	26.17 (1.95)	25.03 (2.53)	−	−	−	1.70 (1.88)	1.63 (2.65)	0.911
FAB													
Similarities	2.57 (0.50)	2.33 (0.48)	0.071	2.73 (0.45)	2.43 (0.57)	2.80 (0.41)	2.57 (0.50)	0.17 (0.38)	0.10 (0.48)	0.553	0.23 (0.43)	0.23 (0.57)	1.000
Lexical fluency	2.80 (0.41)	2.50 (0.63)	0.032	2.70 (0.53)	2.60 (0.56)	2.83 (0.38)	2.80 (0.41)	−0.10 (0.55)	0.10 (0.61)	0.186	0.03 (0.49)	0.30 (0.53)	0.049
Motor series	2.37 (0.85)	2.40 (0.89)	0.883	2.60 (0.77)	2.73 (0.64)	2.47 (0.86)	2.90 (0.40)	0.23 (0.86)	0.33 (0.99)	0.678	0.10 (1.03)	0.50 (1.04)	0.140
Conflicting instructions	2.83 (0.59)	2.73 (0.78)	0.58	2.80 (0.66)	2.90 (0.55)	2.83 (0.59)	2.77 (0.77)	−0.03 (0.89)	0.17 (0.99)	0.413	0.00 (0.26)	0.03 (1.07)	0.869
Go–No Go	2.67 (0.55)	2.40 (0.93)	0.182	2.43 (0.82)	2.50 (0.68)	2.53 (0.73)	2.17 (0.99)	−0.23 (0.86)	0.10 (0.88)	0.144	−0.13 (0.94)	−0.23 (1.22)	0.723
Prehension behavior	3.00 (0.00)	3.00 (0.00)	-	3.00 (0.00)	3.00 (0.00)	3.00 (0.00)	3.00 (0.00)	0.00 (0.00)	0.00 (0.00)	−	0.00 (0.00)	0.00 (0.00)	−
Total score	16.23 (1.48)	15.37 (1.25)	0.017	16.27 (1.60)	16.17 (1.18)	16.47 (1.28)	16.20 (1.54)	0.03 (1.81)	0.80 (1.30)	0.064	0.23 (1.45)	0.83 (1.66)	0.142
JART													
Total number of correct answers	21.47 (3.41)	18.80 (5.44)	0.027	21.67 (3.78)	19.57 (5.35)	22.33 (3.41)	20.50 (4.77)	0.20 (1.03)	0.77 (1.50)	0.094	0.87 (1.43)	1.70 (2.12)	0.080
Full scale IQ	112.87 (7.00)	107.47 (11.17)	0.029	113.30 (7.67)	109.00 (11.01)	114.67 (6.81)	110.93 (9.72)	0.43 (2.06)	1.53 (3.00)	0.104	1.80 (2.80)	3.47 (4.34)	0.082
Verbal IQ	114.77 (8.04)	108.90 (12.38)	0.034	115.40 (8.68)	110.57 (12.22)	116.87 (7.85)	112.70 (10.88)	0.63 (2.37)	1.67 (3.46)	0.182	2.10 (3.16)	3.80 (4.66)	0.103
Performance IQ	108.80 (5.22)	104.97 (8.15)	0.034	109.27 (5.58)	106.13 (7.93)	110.23 (5.00)	107.50 (7.02)	0.47 (1.57)	1.17 (2.23)	0.165	1.43 (2.08)	2.53 (3.17)	0.117
WMSR													
Verbal memory	59.60 (15.22)	53.43 (12.53)	0.092	68.80 (14.50)	58.07 (11.46)	75.20 (17.55)	64.73 (11.63)	10.97 (14.84)	7.93 (12.99)	0.403	15.60 (12.91)	11.30 (9.77)	0.151
Visual memory	55.53 (6.62)	51.33 (9.64)	0.054	57.63 (6.71)	54.63 (7.88)	60.23 (4.79)	56.63 (6.78)	9.20 (9.93)	4.63 (11.10)	0.098	4.70 (6.80)	5.30 (6.75)	0.733
General memory	115.47 (17.83)	104.77 (18.62)	0.027	126.43 (16.44)	112.70 (15.56)	135.43 (19.65)	121.37 (15.60)	2.10 (8.81)	3.30 (5.61)	0.532	19.97 (16.00)	16.60 (12.49)	0.367
Attention/Concentration	59.13 (7.01)	57.83 (7.40)	0.488	60.70 (8.63)	57.73 (6.34)	60.97 (6.64)	57.60 (7.26)	1.57 (7.17)	−0.10 (5.38)	0.313	1.83 (6.72)	−0.23 (6.17)	0.220
GDS													
Total score	2.83 (2.72)	1.57 (2.94)	0.089	2.27 (2.52)	1.73 (3.07)	2.37 (3.18)	1.73 (3.31)	−0.57 (2.92)	0.17 (1.51)	0.227	−0.47 (3.61)	0.17 (2.56)	0.436
Stroop													
Step1	53.93 (8.45)	50.23 (8.39)	0.094	56.53 (6.48)	49.87 (9.27)	57.93 (7.93)	52.23 (8.24)	2.60 (6.09)	−0.37 (4.07)	0.031	4.00 (4.39)	2.00 (4.23)	0.078
Step2	44.73 (10.76)	42.53 (8.25)	0.378	47.73 (6.81)	42.90 (8.18)	47.07 (10.54)	42.93 (7.32)	3.00 (8.99)	0.37 (3.99)	0.148	2.33 (9.24)	0.40 (4.52)	0.307
Step3	36.47 (5.37)	32.93 (5.98)	0.019	36.73 (5.64)	34.17 (5.52)	37.80 (5.80)	34.23 (6.25)	0.27 (2.77)	1.23 (3.72)	0.258	1.33 (4.31)	1.30 (3.72)	0.975
Step4	31.60 (4.85)	26.97 (8.58)	0.013	32.63 (6.08)	28.37 (6.33)	33.57 (6.06)	28.93 (6.45)	1.03 (3.82)	1.40 (5.99)	0.778	1.97 (4.61)	1.97 (4.73)	1.000
Stroop interference rate	16.25 (19.07)	15.07 (10.21)	0.766	15.53 (7.75)	13.42 (9.70)	19.55 (14.84)	17.41 (9.56)	−0.72 (18.04)	−1.65 (8.76)	0.800	3.30 (21.78)	2.34 (9.26)	0.826
Reverse− Stroop interference rate	12.76 (10.18)	19.39 (17.21)	0.075	10.93 (10.78)	17.06 (12.53)	10.31 (14.32)	15.46 (10.75)	−1.83 (11.45)	−2.33 (17.08)	0.896	−2.45 (16.37)	−3.93 (17.29)	0.736
WAIS-IV													
Perceptual reasoning index	87.43 (11.95)	82.60 (10.54)	0.102	93.20 (13.49)	89.43 (11.38)	95.67 (12.39)	88.90 (9.03)	5.77 (8.34)	6.83 (8.49)	0.625	8.23 (8.28)	6.30 (7.96)	0.360
Working memory index	93.20 (9.07)	87.20 (9.36)	0.014	94.17 (10.54)	88.63 (9.11)	99.17 (11.67)	90.20 (10.92)	0.97 (9.86)	1.43 (6.70)	0.831	5.97 (6.86)	3.00 (7.00)	0.103
Processing speed index	113.13 (10.98)	106.57 (10.64)	0.022	114.77 (10.26)	107.53 (11.94)	116.87 (12.09)	111.30 (11.36)	1.63 (6.69)	0.97 (8.51)	0.737	3.73 (8.25)	4.73 (8.49)	0.645
D-CAT													
1 digit total	277.77 (57.11)	257.40 (62.39)	0.192	305.00 (57.75)	279.77 (65.01)	302.87 (61.07)	288.23 (60.68)	27.23 (26.18)	22.37 (50.04)	0.639	25.10 (39.00)	30.83 (44.05)	0.596
2 digits total	236.17 (34.49)	232.77 (42.73)	0.736	236.17 (35.04)	232.90 (44.38)	242.97 (35.10)	232.70 (46.83)	0.00 (25.29)	0.13 (24.39)	0.983	6.80 (24.07)	−0.07 (26.73)	0.300
3 digits total	181.20 (30.72)	176.77 (35.82)	0.609	188.23 (36.20)	173.40 (31.43)	191.77 (37.61)	180.03 (37.84)	7.03 (17.84)	−3.37 (23.73)	0.060	10.57 (25.78)	3.27 (29.99)	0.316
1 digit miss	0.02 (0.04)	0.02 (0.03)	0.723	0.02 (0.04)	0.03 (0.04)	0.03 (0.05)	0.03 (0.05)	0.00 (0.05)	0.01 (0.05)	0.294	0.01 (0.03)	0.01 (0.05)	0.500
2 digits miss	0.06 (0.05)	0.08 (0.05)	0.194	0.05 (0.04)	0.07 (0.05)	0.06 (0.06)	0.08 (0.07)	−0.01 (0.05)	−0.01 (0.06)	0.833	0.00 (0.05)	0.00 (0.08)	0.933
3 digits miss	0.10 (0.07)	0.11 (0.07)	0.473	0.08 (0.07)	0.09 (0.06)	0.08 (0.07)	0.10 (0.08)	−0.02 (0.07)	−0.02 (0.07)	0.949	−0.01 (0.07)	−0.01 (0.08)	0.676
Rate of change (2digits)	0.87 (0.15)	0.93 (0.18)	0.14	0.78 (0.08)	0.85 (0.12)	0.82 (0.10)	0.81 (0.08)	−0.09 (0.12)	−0.08 (0.16)	0.952	−0.05 (0.14)	−0.12 (0.16)	0.093
Rate of change (3digits)	0.66 (0.12)	0.71 (0.16)	0.211	0.62 (0.09)	0.64 (0.12)	0.64 (0.09)	0.63 (0.10)	−0.04 (0.07)	−0.08 (0.15)	0.275	−0.03 (0.12)	−0.08 (0.15)	0.111

## Data Availability

Data is contained within the article or Supplementary Material.

## Supplementary Materials

The following supporting information can be downloaded at: https://www.mdpi.com/article/10.3390/nu15061499/s1, Table S1: Dietary ingredients consumed in the usual diet.
