# Supplementary material for: Supplementation with Flaxseed Oil Rich in Alpha-Linolenic Acid Improves Verbal Fluency in Healthy Older Adults
Source: Nutrients. 2023 Mar 21;15(6):1499. doi: 10.3390/nu15061499 (PMC10056498; doi:10.3390/nu15061499)
Supplement: Supplementary file 1 [file nutrients-15-01499-s001.zip › Table S1.pdf]

Table S1. Dietary ingredients consumed in the usual diet.

Dietary ingredients	Unit	Control	ALA	*p value*
pre-intervention				
energy	kcal	1768.73 (366.18)	1912.37 (303.07)	*0.103*
moisture	g	970.51 (247.50)	1064.31 (238.28)	*0.140*
protein	g	65.81 (15.28)	68.76 (16.59)	*0.478*
lipids	g	58.31 (17.60)	58.51 (16.37)	*0.963*
Carbohydrates	g	232.97 (52.17)	265.80 (37.12)	*0.007*
Ash content	g	17.01 (4.44)	18.13 (4.45)	*0.334*
Sodium	mg	3800.60 (1187.82)	3967.03 (1017.16)	*0.562*
Potassium	mg	2452.17 (764.11)	2717.57 (774.66)	*0.187*
Calcium	mg	554.07 (185.96)	599.00 (171.97)	*0.335*
magnesium	mg	279.07 (76.94)	294.03 (74.61)	*0.447*
phosphorus	mg	994.13 (251.13)	1072.40 (248.62)	*0.230*
iron	mg	7.82 (1.96)	8.18 (2.06)	*0.483*
zinc	mg	7.47 (1.73)	7.94 (1.45)	*0.259*
Copper	mg	1.16 (0.29)	1.25 (0.25)	*0.226*
manganese	mg	2.69 (0.67)	2.84 (0.55)	*0.364*
iodine	µg	865.93 (564.52)	894.80 (581.00)	*0.846*
Selenium	µg	60.53 (13.29)	59.60 (19.05)	*0.827*
Chromium	µg	7.07 (2.10)	7.80 (2.20)	*0.192*
Molybdenum	µg	161.57 (46.38)	181.63 (38.04)	*0.072*
Retinol	µg	155.07 (56.26)	180.77 (62.80)	*0.100*
β-carotene	µg	3745.40 (1603.80)	3914.07 (1854.12)	*0.708*
β-carotene equivalent	µg	4335.89 (1857.55)	4540.66 (2089.28)	*0.690*
Retinol activity equivalent	µg	518.77 (193.06)	561.19 (201.41)	*0.408*
Vitamin D	µg	5.05 (2.30)	6.59 (3.59)	*0.053*
alpha-Tocopherol	mg	7.22 (2.11)	7.59 (2.48)	*0.540*
vitamin K	µg	271.17 (90.46)	272.66 (101.39)	*0.952*
Vitamin B1	mg	1.00 (0.28)	0.99 (0.27)	*0.889*
Vitamin B2	mg	1.10 (0.31)	1.20 (0.41)	*0.286*
Niacin	mg	14.02 (4.16)	14.87 (5.03)	*0.480*
Niacin Equivalent	mg	26.62 (6.73)	28.02 (7.74)	*0.457*
Vitamin B6	mg	1.19 (0.36)	1.29 (0.38)	*0.313*
Vitamin B12	µg	5.45 (2.11)	6.68 (3.40)	*0.096*
Folic acid	µg	310.89 (103.36)	329.99 (102.56)	*0.475*
Pantothenic acid	mg	5.48 (1.43)	5.92 (1.33)	*0.225*
Biotin	µg	31.32 (8.94)	32.39 (6.82)	*0.605*
Vitamin C	mg	96.13 (41.23)	107.03 (43.35)	*0.322*
Saturated fatty acids	g	18.06 (6.01)	18.62 (5.68)	*0.715*
Monounsaturated fatty acid	g	20.45 (6.78)	19.88 (5.94)	*0.730*
Polyunsaturated fatty acids	g	12.98 (3.76)	12.73 (4.06)	*0.805*
cholesterol	mg	296.33 (92.90)	315.27 (103.24)	*0.458*
Soluble dietary fibre	g	3.51 (1.05)	3.56 (1.14)	*0.851*
Insoluble dietary fibre	g	10.53 (3.19)	10.96 (3.31)	*0.610*
Total dietary fibre	g	14.90 (4.53)	15.52 (4.55)	*0.599*
Salt equivalent.	g	9.60 (3.03)	10.00 (2.57)	*0.580*
Alcohol content	g	5.56 (10.04)	5.23 (7.33)	*0.885*
Total fatty acids	g	51.53 (15.77)	51.26 (14.68)	*0.947*
n.3 polyunsaturated fatty ac	g	2.16 (0.72)	2.35 (0.87)	*0.354*
n.6 polyunsaturated fatty ac	g	10.80 (3.15)	10.34 (3.33)	*0.587*

Table S1. Cont.

Dietary ingredients	Unit	Control	ALA	*p value*
post-intervention				
energy	kcal	1913.03 (423.53)	1933.77 (458.56)	*0.856*
moisture	g	1029.29 (252.44)	1036.27 (217.65)	*0.909*
protein	g	75.14 (16.88)	73.42 (23.45)	*0.745*
lipids	g	65.71 (21.63)	65.62 (23.16)	*0.988*
Carbohydrates	g	244.90 (59.52)	249.76 (52.12)	*0.738*
Ash content	g	19.86 (5.17)	18.77 (5.25)	*0.418*
Sodium	mg	4560.70 (1257.40)	4183.97 (1511.30)	*0.298*
Potassium	mg	2750.83 (802.67)	2690.00 (699.68)	*0.755*
Calcium	mg	643.70 (207.81)	652.73 (205.62)	*0.866*
magnesium	mg	313.70 (79.85)	302.50 (83.61)	*0.598*
phosphorus	mg	1139.47 (286.52)	1135.47 (321.21)	*0.960*
iron	mg	9.02 (2.13)	8.55 (2.43)	*0.429*
zinc	mg	8.34 (1.92)	8.23 (2.31)	*0.837*
Copper	mg	1.23 (0.30)	1.21 (0.28)	*0.795*
manganese	mg	2.81 (0.70)	2.68 (0.53)	*0.413*
iodine	μg	1200.53 (640.03)	998.47 (554.21)	*0.196*
Selenium	μg	68.10 (15.99)	64.90 (21.67)	*0.518*
Chromium	μg	7.77 (2.31)	7.67 (2.37)	*0.869*
Molybdenum	μg	171.33 (48.13)	173.73 (43.06)	*0.839*
Retinol	μg	195.60 (77.32)	203.93 (79.32)	*0.682*
β-carotene	μg	4399.97 (1482.39)	3983.07 (1339.44)	*0.258*
β-carotene equivalent	μg	5023.65 (1692.77)	4559.56 (1503.11)	*0.266*
Retinol activity equivalent	μg	616.82 (188.39)	586.34 (182.12)	*0.527*
Vitamin D	μg	7.07 (2.74)	7.18 (3.72)	*0.894*
alpha-Tocopherol	mg	8.07 (2.42)	7.90 (2.53)	*0.791*
vitamin K	μg	310.67 (89.10)	286.93 (85.03)	*0.295*
Vitamin B1	mg	1.12 (0.31)	1.04 (0.37)	*0.411*
Vitamin B2	mg	1.37 (0.49)	1.30 (0.47)	*0.591*
Niacin	mg	16.52 (4.40)	15.89 (6.48)	*0.661*
Niacin Equivalent	mg	30.69 (7.34)	29.79 (10.43)	*0.698*
Vitamin B6	mg	1.35 (0.37)	1.29 (0.39)	*0.543*
Vitamin B12	μg	7.36 (2.75)	7.27 (3.48)	*0.915*
Folic acid	μg	359.87 (104.20)	331.21 (82.35)	*0.242*
Pantothenic acid	mg	6.14 (1.62)	6.12 (1.71)	*0.959*
Biotin	μg	34.93 (10.44)	33.96 (8.81)	*0.699*
Vitamin C	mg	103.43 (40.83)	96.63 (25.99)	*0.445*
Saturated fatty acids	g	20.78 (7.76)	21.25 (8.10)	*0.818*
Monounsaturated fatty acid	g	22.67 (7.92)	22.39 (8.30)	*0.893*
Polyunsaturated fatty acids	g	14.01 (4.58)	13.81 (4.98)	*0.868*
cholesterol	mg	357.90 (119.86)	350.33 (139.56)	*0.823*
Soluble dietary fibre	g	3.73 (1.07)	3.43 (0.82)	*0.240*
Insoluble dietary fibre	g	11.39 (3.42)	10.61 (2.54)	*0.318*
Total dietary fibre	g	16.47 (4.66)	15.22 (3.60)	*0.250*
Salt equivalent.	g	11.52 (3.18)	10.57 (3.84)	*0.299*
Alcohol content	g	4.55 (6.15)	5.40 (7.07)	*0.621*
Total fatty acids	g	57.51 (19.33)	57.49 (20.48)	*0.996*
n.3 polyunsaturated fatty ac	g	2.50 (0.91)	2.54 (1.00)	*0.852*
n.6 polyunsaturated fatty ac	g	11.49 (3.76)	11.23 (4.06)	*0.803*

(mean (SD))
